# A novel model of common Toll-like receptor 4- and injury-induced transcriptional themes in human leukocytes

**DOI:** 10.1186/cc9283

**Published:** 2010-10-07

**Authors:** Beatrice Haimovich, Michael T Reddell, Jacqueline E Calvano, Steve E Calvano, Marie A Macor, Susette M Coyle, Stephen F Lowry

**Affiliations:** 1 Department of Surgery, Division of Surgical Sciences, UMDNJ-Robert Wood Johnson Medical School, New Brunswick, New Jersey, USA

## Abstract

**Introduction:**

An endotoxin challenge, sepsis, and injury/trauma, trigger significant changes in human peripheral blood leukocytes (PBL) gene expression. In this study, we have sought to test the hypothesis that the Toll-like receptor 4 (TLR4) induced transcription patterns elicited in humans exposed to *in vivo *endotoxin would parallel gene expression patterns observed in trauma patients with initial non-infectious injury. In addition, we sought to identify functional modules that are commonly affected by these two insults of differing magnitude and duration.

**Methods:**

PBL were obtained from seven adult human subject experimental groups. The groups included a group of healthy, hospitalized volunteers (*n *= 15), that comprised four study groups of subjects challenged with intravenous endotoxin, without or with cortisol, and two serial samplings of trauma patients (*n *= 5). The PBL were analyzed for gene expression using a 8,793 probe microarray platform (Gene Chip^® ^Focus, Affymetrix). The expression of a subset of genes was determined using qPCR.

**Results:**

We describe sequential selection criteria of gene expression data that identifies 445 genes that are significantly differentially expressed (both *P *≤ 0.05 and >1.2 fold-change) in PBL derived from human subjects during the peak of systemic inflammatory responses induced by *in vivo *endotoxin, as well as in PBL obtained from trauma patients at 1 to 12 days after admission. We identified two functional modules that are commonly represented by this analysis. The first module includes more than 50 suppressed genes that encode ribosomal proteins or translation regulators. The second module includes up-regulated genes encoding key enzymes associated with glycolysis. Finally, we show that several circadian clock genes are also suppressed in PBL of surgical ICU patients.

**Conclusions:**

We identified a group of >400 genes that exhibit similar expression trends in PBL derived from either endotoxin-challenged subjects or trauma patients. The suppressed translational and circadian clock modules, and the upregulated glycolytic module, constitute a robust and long lasting PBL gene expression signature that may provide a tool for monitoring systemic inflammation and injury.

## Introduction

Circulating leukocytes play a central role in host immunity, and are a major source of inflammatory mediators released in response to exposure to pathogen-associated molecular pattern(s) (PAMPs), such as endotoxin [1,2]. Gene expression profiling of human peripheral blood leukocytes (PBL) or mononuclear cells, have revealed robust gene expression changes that are detectable within two hours of an *in vivo *endotoxin challenge [3,4]. This abbreviated model of acute, Toll-like receptor 4 (TLR4) induced inflammation exhibits a return to baseline for nearly all systemic and cellular perturbations within 24 hours [3-5]. Genome-wide analysis of network-based classifications of PBL gene expression data have demonstrated significant changes in the transcriptional expression of genes associated with several pathways and cellular functions, including pathogen recognition and immune responses, metabolism, bioenergetics, translation, and transcription [3,4,6,7].

Studies in animal models have highlighted that TLR4 signaling is initiated not only by PAMPs, but also by damage-associated molecular patterns (DAMPs) that are released by host tissues when exposed to more extreme stress conditions, such as injury and infection (for example, [8-10]). High-mobility group box 1 (HMGB1), and heat shock proteins (HSP) HSP-70 and HSP-90, are examples of DAMPs that signal through TLR4 [1,11-13]. In addition, there is evidence that cellular reactive oxygen species (ROS) may also engage TLR4 and activate TLR-dependent signaling events [14,15]. Collectively, these data imply that endogenous DAMPs and ROS, as well as endotoxin or other PAMPs, have the capacity to initiate common, TLR4-related signaling cascades.

Building on this concept, we hypothesized that the TLR4 induced transcription patterns elicited by *in vivo *endotoxin exposure would parallel gene expression patterns observed in patients with initial non-infectious injury. In this preliminary analysis, we identified a group of 445 genes that exhibited similar expression trends in PBL in both endotoxin-challenged subjects and trauma patients. While these changes in TLR4 induced gene expression are short-lived in lipopolysaccharide (LPS) challenged subjects, the patterns observed after injury persist for up to 12 days after trauma. Included in this group are multiple downregulated genes that are associated with the translational apparatus, as well as several upregulated genes, which encode proteins exhibiting a key role in glycolysis. Consistent with the known acute effect of endotoxin [16], we also document that the expression of several circadian clock genes is suppressed in PBL from such patients. These observations identify common TLR4/injury induced transcriptional themes that exist in PBL during systemic inflammation and trauma.

## Materials and methods

### Volunteer subjects

Healthy adult subjects were recruited by public advertisement and screened for inclusion in this study under approved guidelines of the Institutional Review Board of the Robert Wood Johnson Medical School. Written informed consent was obtained from all patients participating in the study. Inclusion criteria for the study were normal general health as demonstrated by medical history and physical examination, complete blood count, and basic metabolic panel within normal lab limits. Exclusion criteria included a history of any acute or chronic disease, arrhythmia, recent history of alcohol, drug or medication ingestion, pregnancy or prior exposure to endotoxin in the experimental setting.

Upon accrual to the study, the subjects were admitted to the Clinical Research Center (CRC) at UMDNJ-Robert Wood Johnson Medical School the afternoon prior to the study and a repeat examination confirmed that no changes in health status had occurred since enrollment. Female subjects underwent a urine pregnancy test. The subjects' characteristics are summarized in Table 1. The volunteer subjects were placed nil per os (NPO) at midnight prior to the endotoxin study day, and underwent intravenous fluid hydration (1 ml/kg-hr) until completion of the acute study phase. Following admission, subjects were randomized to one of two study groups. Subjects assigned to Groups B and D (Table 2) received a placebo infusion of physiologic saline prior to endotoxin administration. PBL samples obtained from these subjects prior to endotoxin infusion were used as baseline (Group A; Table 2). Subjects assigned to Groups C and E (Table 2) received continuous intravenous infusion of cortisol (3 μg/kg/min) for 12 hours starting six hours before endotoxin administration [17]. Subjects assigned to Groups B to E received a one-time intravenous dose (2 ng/kg) of endotoxin (NIH Clinical Center Reference Endotoxin; CC-RE-Lot2) at 0 hour (0900 clock time). Blood samples were drawn at six hours (Groups B and C; Table 2) and 24 hours (Groups D and E; Table 2) post-endotoxin.

**Table 1:** Volunteer subject and patient characteristics

Subject characteristics		
	Volunteers	Patients
n =	15	5
Age^a^	24 ± 2	31 ± 7
Age Range	18 to 36	19 to 54
Male/Female	9/5	4/1
SICU LOS		19 ± 6
SICU LOS range		9 to 40
Hospital LOS		32 ± 6
Hospital LOS range		26 to 57
Admission APACHE II		20 ± 2
APACHE II Range		14 to 28
Injury Severity Score		29 ± 5 (range: 9 to 50)
Transfusion^b^		4 ± 2 (range: 0 to 14)

^a ^Means ± standard errors of the means.

^b ^Two patients received more than five units of RBC.

APACHE II, Acute Physiology and Chronic Health Evaluation II; LOS, length of stay.

**Table 2:** Volunteer subjects and ICU patient samples classification

Group		Sample numbers
A	Baseline (control)	4
B	Six hours endotoxin	7
C	Six hours cortisol plus endotoxin	7
D	24 hours endotoxin	5
E	24 hours cortisol plus endotoxin	6
F	Surgical ICU patients ≤5 days post-admission	5
G	Surgical ICU patients ≤12 days post-admission	4

PBL samples were obtained from volunteer subjects who were administered saline alone, (Group A), saline plus endotoxin (Groups B and D), or cortisol plus endotoxin (Groups C and E), as detailed in the Materials and methods section. PBL samples obtained from surgical ICU patients ≤5 days, and ≤12 days post-admission were classified in Groups F and G, respectively.

### Patients

Patients were accrued from the adult Surgical ICU at Robert Wood Johnson University Hospital under a protocol approved by the Institutional Review Board of the Robert Wood Johnson Medical School.

The patient demographic characteristics are described in Table 1. An anticipated ICU stay of at least 72 hours and anticipated ultimate survival were utilized as inclusion criteria. Patients were excluded if they had a suspected or confirmed infection, received an organ transplant, required more than six units of blood transfusions and/or had severe traumatic brain injury (admitting GCS < 8). Blood samples were first drawn within one to five days of ICU admission, and again five to seven days later.

Blood samples were drawn in EDTA tubes, and centrifuged at 400 × g for 10 minutes. The plasma was removed, and the red blood cell/leukocyte pellet was treated with bicarbonate-buffered ammonium chloride lysing solution (0.1% potassium bicarbonate; 0.826% ammonium chloride in H_2_0) at a ratio of 1 part red blood cell/leukocytes to 20 parts lysing solution for 15 minutes in order to lyse the red blood cells. The leukocytes were then collected by centrifugation and washed once in lysing solution. After another centrifugation, a small aliquot of the leukocyte pellet was removed for performing a flow cytometric differential cell count on the healthy subjects. The leukocyte pellet was lysed in TRIzol™ solution (Sigma, St. Louis, MO, USA), sheared 10 times with an 18-gauge needle, and frozen at -70°C.

### Preparation of RNA, cDNA, and labeled cRNA

#### Total RNA

Cell lysates in TRIzol™ (Sigma) were thawed and treated with chloroform. The RNA was isolated from the aqueous phase and precipitated with isopropyl alcohol. Following washing with alcohol, the RNA pellet was dried and dissolved in DEPC water. The quality and quantity of the isolated RNA was evaluated using the 2100 Bioanalyzer™ (Agilent Technologies, Palo Alto, CA, USA).

#### cDNA synthesis

First strand cDNA synthesis was performed using reverse transcription (SuperScriptII, Invitrogen, Carlsbad, CA, USA) in a reaction containing 5 μg of total RNA, T7-oligo (dt)_24 _primer, DTT, and dNTP mix. Second strand cDNA synthesis was then carried out by reaction of the first strand with DNA polymerase I, DNA ligase, and dNTP mix, followed by additional reaction with T4 DNA polymerase (Invitrogen). Double-stranded cDNA was purified using the GeneChip Sample Cleanup Module (Affymetrix, Santa Clara, CA, USA).

#### cRNA synthesis

Biotinylated cRNA was synthesized from the double-stranded cDNA using GeneChip expression 3'-amplification reagents for IVT labeling (Affymetrix). This reaction uses MEGAscript T7 polymerase in the presence of a mixture of the four natural ribonucleotides and one biotin-conjugated analog. The biotinylated cRNA so-generated was then cleaned up using the GeneChip Sample Cleanup Module (Affymetrix).

### Microarray analysis

Steps outlined in this section were performed by the microarray core facility at this institution. Following fragmentation of the biotinylated cRNA, 15 μg was placed in hybridization cocktail, heated to 95°C, centrifuged and then hybridized to the Focus™ GeneChip microarray (Affymetrix) for 16 hours at 45°C. Chips were then washed, stained with streptavidin phycoerythrin and scanned on the Agilent Gene Array Scanner™ (Agilent Technologies).

### Analysis of microarray data

We compiled a database that includes 38 Focus GeneChip^® ^microarrays (Affymetrix) derived from the study groups outlined in Table 2. The microarray data have been submitted to Gene Expression Omnibus [GEO:GSE22278]. The database includes two matching PBL samples obtained from five patients (Table 2). For four out of the five patients, the blood samples were obtained within 5 days (Group F) and 12 days of admission (Group G). The fifth patient was also sampled in the later phase but the microarray displayed a background level that precluded statistical analysis.

Focus Gene chip data CEL files were imported, grouped, and analyzed using GeneSpring™ software (Agilent Technologies). Primary analysis was carried out by log2 transformation followed by transformation to the median and RMA (quantile) normalization. Advanced significance analysis was performed on normalized-transformed data utilizing unpaired Student's *t*-tests. We further defined significantly expressed probes as those with a *P*-value < 0.05 and ≥1.2-fold change from baseline. Data were also exported for analysis by Ingenuity Pathway Analysis ™ (Ingenuity, Palo Alto, CA, USA) as previously described [3].

### qPCR

Where indicated, RNA was extracted as described above and reversed transcribed to cDNA using High capacity cDNA Archive kit™ (Applied Biosystems, Foster City, CA, USA). Gene expression was analyzed in duplicate by quantitative real-time polymerase chain reaction (qPCR) using inventoried TaqMan^® ^gene expression assays (Applied Biosystems) as described [16]. A list of the gene expression assays can be found in [16]. The relative gene expression analysis was performed using the 2^-ΔΔCT ^method [18]. The level of *beta-2-microglobulin (B2M) *expression was used as an internal reference [3,19,20].

## Results and discussion

### Differential gene expression in PBL derived from *in vivo *endotoxin challenged subjects and trauma patients

**Figure 1:**
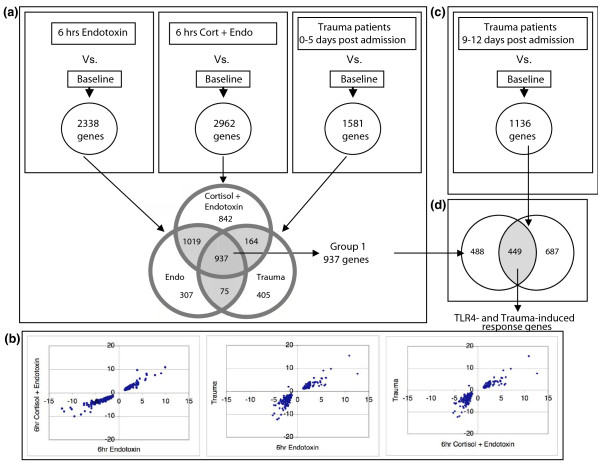
**TLR4 and injury responsive (TIR) genes selection criteria**. **(a) **Genes that were significantly differentially expressed (*P- *value < 0.05 and ≥1.2-fold change) in PBL obtained from subjects challenged with *in vivo *endotoxin (Endo) for six hours (2 ng/kg), subjects infused with cortisol (Cort) (3 μg/kg/min) for 12 hours starting 6 hours before endotoxin administration (Cort + Endo), or from trauma patients PBL obtained within the initial five days after ICU admission, as compared to baseline, were identified. The Venn diagram identifies the genes that are common between groups. Nine hundred thirty-seven (937) genes were common to all three groups. **(b) **Scatter plot analysis comparing Group 1 genes expression trends between the indicated groups. **(c) **Genes that were significantly differentially expressed in trauma patients PBL obtained within 9 to 12 days after ICU admission as compared to baseline were identified. **(d) **Four hundred and forty-five genes were differentially expressed in both *in vivo *endotoxin challenged PBL and in PBL obtained from trauma patients over a period of 1 to 12 days after admission.

Prior studies [3,4] indicated a maximal change in PBL gene expression at the six-hour time point post endotoxin infusion in all volunteer subjects. Hence, this time-point was chosen to depict the influence of endotoxin. Expressed gene selection proceeded from the array database as outlined in Figure 1. Arrays representing PBL obtained after an *in vivo *endotoxin challenge (Group B), or antecedent cortisol plus endotoxin challenge (Group C), as well as those obtained from trauma patients within five days of admission (Group F), were independently compared to baseline. Gene probes that were significantly differentially expressed (both *P *≤ 0.05 and >1.2 fold-change) were then selected (Figure 1a). Out of the 8,793 genes represented on the Focus GeneChip^® ^(Affymetrix) microarrays, 2,338 (27%) and 2,962 (34%) genes were differentially expressed, by the criteria described above, in PBL six hours after challenge with endotoxin, without or with cortisol, as compared to baseline (Figure 1a). Of these, 1,956 were common to PBL treated with endotoxin (Group B) and cortisol plus endotoxin (Group C) (Figure 1a).

Numerous genes (1,581; 18%) were also differentially expressed (both *P *≤ 0.05 and >1.2 fold-change) in PBL obtained from trauma patients within the first five days of admission as compared to baseline values of normal subjects. Based on these similarities, 937 genes were significantly differentially expressed in all three groups (Group 1; Figures 1a). Scatter plot analyses revealed that the gene expression trends were highly correlated among the three groups (Figure 1b). These data suggest a significant commonality among differentially expressed genes during the early, dynamic phase of TLR4-induced inflammation resulting from endotoxin infusion, and those differentially expressed in PBL in the early post-trauma time period.

### Differential gene expression in PBL during prolonged injury

Next, we sought to determine which of the 937 genes that are differentially expressed during the peak of systemic inflammatory responses, and during the first several days after a trauma event, remain differentially expressed in PBL obtained at later time points of up to 12 days after ICU admission. To that end, we first selected 1,136 genes that were differentially expressed in PBL obtained from trauma patients after 9 to 12 days of admission (Figure 1c), and then identified genes that were common to both this later injury phase group and those genes defined as Group 1 genes (Figure 1d). This resulted in the identification of 445 genes (5.4%) that persisted in differential expression in response to TLR4-induced systemic inflammation and/or injury. We refer to this group of TLR4 and injury responsive genes as "TIR" genes. The 445 TIR genes are listed in Table S1, which can be found in Additional file 1.

**Figure 2:**
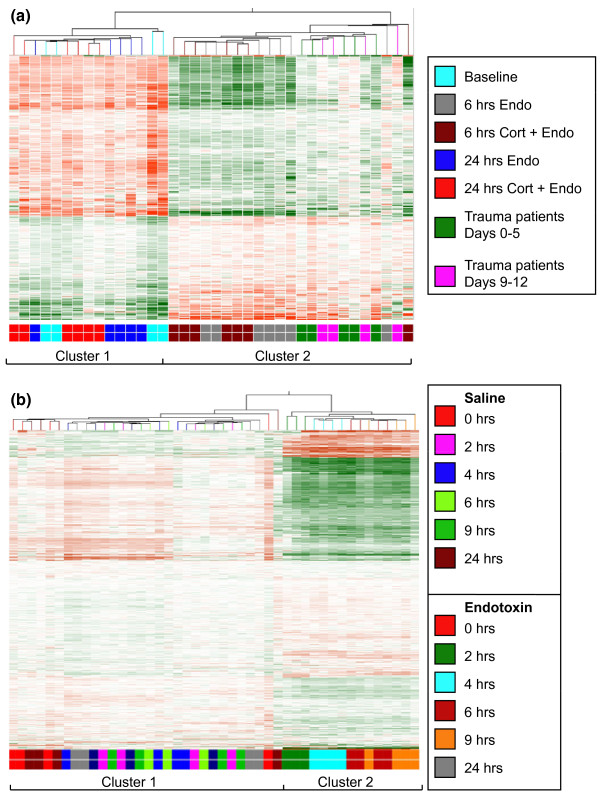
**Clustering analysis of TLR4 and injury responsive (TIR) genes**. **(a) **The panel depicts hierarchical cluster analysis of the 445 TIR genes selected from 38 Gene Chip^® ^Focus Array database described in Table 2. **(b) **The panel depicts hierarchical cluster analysis of TIR genes selected from a 45 Hu133B^® ^Array database described in [3]. Due to probe replicates, the 445 TIR genes are represented by a total of 823 probes sets.

The TIR genes selected as outlined in Figure 1, plus those from the 24 hours post-endotoxin groups (Table 2; Groups D and E) were subjected to hierarchical cluster analysis. As shown in Figure 2a, the clustering analysis defined two dominant groups. Cluster 1 included both baseline samples and all PBL samples derived from subjects at 24 hours after endotoxin. Cluster 2 included all the PBL samples derived from subjects at 6 hours post-endotoxin challenge as well as the trauma patient samples.

One strength of the present analysis is the identification of gene expression patterns common to both *de novo *endotoxin and injury-induced conditions. As a consequence, there is likely to be a lesser transcriptional influence of clinical management factors, such as prior transfusions of blood products, vasopressor use, or opiates and other therapeutics since these agents were not utilized in the endotoxin challenged subjects. While we cannot completely exclude interacting effects from interventions and therapies, the common transcriptional themes arising from the present analysis strongly suggest pathways dominated by endotoxin or other TLR4 agonist influences *in vivo*. Although it is documented that circulating endotoxin is frequently detectable in trauma/burn patients [21,22] as well as in more heterogeneous ICU populations [23], the presence of detectable endotoxin is far from uniform in these patients. Since we did not measure endotoxin or other soluble factors, such as HMGB1, S100A/B, or acute phase proteins that may also serve as TLR4 activating ligands, we cannot further speculate on whether the derived leukocyte transcriptional signatures are attributable to endotoxin or other mediators.

We also examined the TIR gene expression trends using a published database [3] [GEO:GSE3284] that includes microarray data derived from four previously reported endotoxin challenged subjects at 0, 2 4, 6, 9 and 24 hours post challenge, and four control subjects studied at parallel time points. The TIR genes showed a robust response in all endotoxin-challenged subjects, and a return to baseline by 24 hours post treatment (Figure 2b). Furthermore, hierarchical cluster analysis revealed two dominant clusters. Cluster 1 included a total of 30 samples representing 26 control samples plus 4 PBL samples obtained from endotoxin challenged subjects at 24 hours post-infusion (Figure 2b). Cluster 2 included all the PBL samples obtained between two and nine hours post-infusion (Figure 2b). This significant degree of correspondence between a prior endotoxin challenged population and the present volunteers group confirms the fidelity of our baseline and endotoxin challenged-subjects analysis.

### TIR genes pathways and interactions

The TIR genes group includes 272 downregulated and 173 upregulated genes (Table S1, which can be found in Additional file 1). The most striking feature of this group of differentially expressed genes is the abundance of *RPL *(ribosomal proteins associated with large 60S ribosomal subunit) and *RPS *genes (ribosomal proteins associated with small 40S ribosomal subunit) (for a recent review see [24]). Furthermore, 50 of the 53 *RPL*/RPS genes are downregulated. Among the downregulated TIR genes are also three *EIF/EEF *genes, which encode translation initiation factors, and six *HNRNP *genes, which regulate pre-mRNA processing and other aspects related to mRNA metabolism (for example, [24,25]).

The expression data were analyzed through the use of Ingenuity Pathway Analysis (Ingenuity^® ^systems) as previously described (for example, [3,26]). This analysis classified the TIR genes into five main modules, each representing 140 genes (the maximum number of genes that the program associates with each module). Three out of the top five modules, which include approximately 230 TIR genes in total, are related to protein synthesis pathways. Two additional pathways, a lipid metabolism pathway, and a cellular assembly and organization pathway, included, respectively, 71- and 68-TIR gene matches.

**Figure 3:**
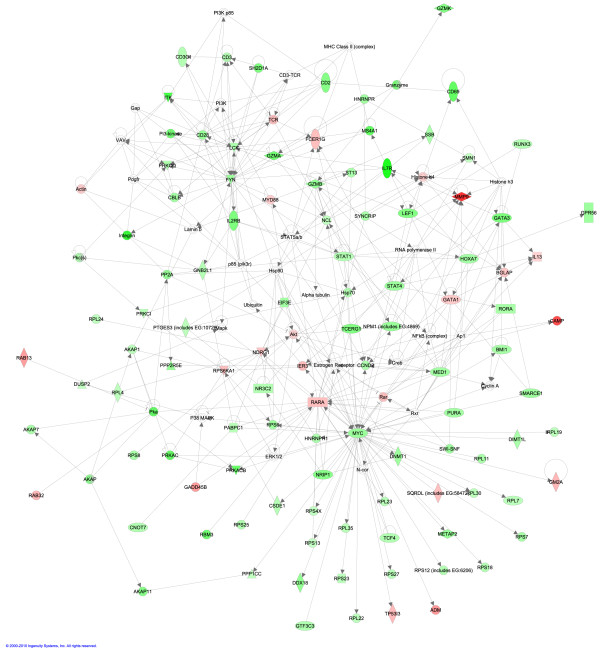
**TLR4 and injury responsive (TIR) genes pathway analysis**. To determine the putative biological role of the TIR genes, the expression data were analyzed through the use of Ingenuity Pathway Analysis. The top ranking module shown in this figure includes 99 TIR genes. *Myc, *depicted on the lower right, is the focal point for the most densely populated node that includes numerous *RPL/RPS *genes.

The top matching module shown in Figure 3 includes 99 TIR genes. *Myc*, a global transcription regulator of many cellular processes, including ribosomal biogenesis and protein synthesis (for example, [24]), is the focal point for the most densely populated node encompassing numerous *RPL/RPS *genes. This large number of suggested interactions is not surprising given that more that 600 genes, including 48 transcription factors, were identified as direct *Myc*-regulated gene targets in human B lymphoid tumor cells alone [27]. Furthermore, TIDBase, a web-based public resource supported by the type 1 diabetes (T1D) research community [28], identified more than 1,400 *Myc*-related interactions. We speculate that the implied reduction of PBL protein synthesis capacity is highly significant. A decline in transcripts associated with transcription was first observed in PBL obtained from endotoxin-challenged subjects [3]. However, the endotoxin-induced changes in PBL gene expression were all transient, with recovery within 24 hours. By contrast, the identification of a similar and persistent gene expression signature in PBL obtained from trauma patients 1 to 12 days post-admission clearly suggests that the translational function of circulating leukocytes is consistently reprogrammed to a lower state.

Importantly, among the upregulated TIR genes were several genes that are known to be associated with glycolysis. These include *PFKFB3*, encoding 6-phosphofructo-2-kinase (PFK-2), and *HK3*, encoding hexokinase 3. PFK-2 is a bifunctional enzyme that catalyzes the synthesis and degradation of fructose 2,6-biphosphate, which in turn, stimulates 6-phosphofructo-1-kinase, the key regulator of mammalian glycolysis [29]. An increase in *PFKFB3 *(also known as *iPFK2*) expression has been documented in endotoxin-treated cultured human monocytes [30]. Hexokinase 3 phosphorylates glucose to produce glucose-6-phosphate, the first intermediate in glycolysis. We also observed an upregulation of *SLC2A3, *encoding the glucose transporter Glut 4, and *PDK3*, encoding pyruvate dehydrogenase kinase (PDK). PDK is an inhibitor of pyruvate dehydrogense complex, which is positioned at the junction between glycolysis and the TCA cycle [31]. In cancer cells, an increase in PDK3 expression was associated with an increase in lactic acid production, which is indicative of a decrease in mitochondrial respiration [32]. These collective changes in gene expression predict an increase in glucose consumption and glycolysis. This possibility is supported by studies in endotoxin-challenged rats, wherein an increase in glucose utilization in multiple organs was observed within hours of an endotoxin or TNFα challenge [33,34]. These data suggest that the systemic conditions induced by acute TLR4 ligation, resulting in enhanced PBL glycolysis, also persist for an extended period after trauma.

**Figure 4:**
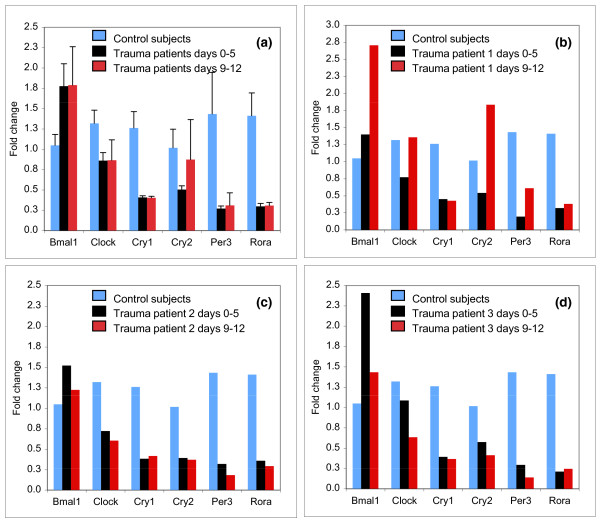
**Clock gene expression in control and surgical ICU patients PBL**. PBL were obtained from four control subjects that received a placebo infusion of physiologic saline and from three ICU patients. The expression of *Bmal1, Clock, Cry1, Cry2, Per3 *and *Rorα *were determined by qPCR. **(a) **Shown are the mean fold change in gene expression observed in PBL obtained from four control subjects and three ICU patients. Error bars are SEM. Two blood samples, referred to as first and second blood draw, were obtained from each patient at a one-week interval. **(b-d) **show the fold change in *Bmal1, Clock, Cry1, Cry2, Per3 *and *Rorα *expression for each of the patients represented in panel A.

Included among the suppressed TIR genes was also *Rorα*, one of the key regulators of the circadian clock [35]. The circadian clock is an autoregulatory feedback network of transcription factors and proteins whose activity and/or availability cycle with a periodicity of approximately 24 h [36-38]. The central "master" clock controlling behavioral circadian rhythms is located in the suprachiasmatic nucleus (SCN) within the brain hypothalamus [39,40]. The central clock both regulates and receives inputs from peripheral clocks present in most tissues, including peripheral blood leukocytes [41-44]. Multiple circadian clock genes, including *Clock, Cry1, Cry2, Per3, *and *Rora*, are significantly suppressed within two hours after an endotoxin-challenge and remain suppressed for up to 17 hours post-infusion [16]. We therefore sought to determine the status of *Clock, Cry1, Cry2, Per3, *and *Rora *expression in a subset of these surgical ICU patient samples. Our analysis revealed a significant and uniform reduction in PBL clock gene expression during the first week of ICU admission (Figure 4). *Bmal1*, the only gene not affected in endotoxin-challenge PBL [16], was also not reduced in PBL obtained from patients. Several genes, including *Cry1, Per3, *and *Rora *remained suppressed in the patients studied for at least an additional week during ICU admission (Figure 4). Our analysis thus suggests that the transient decline in circadian clock gene expression in PBL first noted during systemic inflammation induced by TLR4 activation [16] persists for an extended period in patients with injury induced systemic inflammation.

## Conclusions

Gene-expression profiling has been used to differentiate between disease states, such as a sterile systemic inflammatory syndrome versus early sepsis [45], to define pathways associated with posttraumatic inflammatory responses in the critically ill [26], and to distinguish between gram-positive and gram-negative sepsis, as well as other infectious-ligand induced responses [46-48]. This study describes the identification of a group of 445 genes, which are associated with at least two well-defined biological modules that are dysregulated acutely in response to TLR4 activation and for a prolonged period in response to injury. We also document that the expression of several circadian clock genes is suppressed in PBL from both endotoxin challenged subjects [16] and ICU patients. The expression of this suite of molecular markers may provide a sensitive tool for monitoring patients' state of health.

## Key messages

• We identified a group of 445 PBL genes that are differentially expressed during the peak of TLR4-induced acute systemic inflammation and in trauma patients studied over a 1 to 12 day period after ICU admission.

• The group includes genes associated with translation and glycolysis.

• Several additional genes associated with the circadian clock network are also suppressed in PBL from both endotoxin challenged subjects [16] and ICU patients within 12 days of admission.

• This transcriptional signature may provide a tool for monitoring systemic inflammation and trauma.

## Abbreviations

APACHE II: Acute Physiology and Chronis Health Evaluation II; DAMPs: damage-associated molecular patterns; HMGB1: High-mobility group box 1; HSP: heat shock protein; ICU: intensive care unit; LOS: length of stay; LPS: Lipopolysaccharide; NPO: nil per os (nothing by mouth); PAMPs: pathogen-associated molecular pattern; PBL: peripheral blood leukocytes; ROS: reactive oxygen species; TLR4: Toll like receptor 4.

## Competing interests

The authors declare that they have no competing interests.

## Authors' contributions

BH assisted with the data analysis and prepared the final manuscript. MTR performed all the analysis of gene expression data and pathways. SMC assisted with study design and performance of the clinical studies. JEC performed all microarray studies. MAM recruited all subjects and performed the clinical studies. SEC assisted in study design, while SFL designed the study, oversaw all clinical aspects of the project, assisted with data analysis and prepared the final manuscript.

## Supplementary Material

Additional file 1 **Table S1. TLR4 and injury responsive (TIR) genes list**. All genes included on this list were significantly differentially expressed (*P- *value < 0.05 and ≥1.2-fold change) in PBL obtained from healthy subjects at six hours after challenge with *in vivo *endotoxin, and in trauma patients studied within 1 to 12 days after admission, as compared to baseline healthy subjects (Please see Figure 1 for details). Expression increase relative to baseline is shown in red, and expression decrease is shown in green. Click here for file
