# Supplementary material for: A novel model of common Toll-like receptor 4- and injury-induced transcriptional themes in human leukocytes
Source: Crit Care. 2010 Oct 7;14(5):R177. doi: 10.1186/cc9283 (PMC3219281; doi:10.1186/cc9283)
Supplement: Additional file 1 — Table S1. TLR4 and injury responsive (TIR) genes list. All genes included on this list were significantly differentially expressed (P- value < 0.05 and ≥1.2-fold change) in PBL obtained from healthy subjects at six hours after challenge with in vivo endotoxin, and in trauma patients studied within 1 to 12 days after admission, as compared to baseline healthy subjects (Please see Figure 1 for details). Expression increase relative to baseline is shown in red, and expression decrease is shown in green. [file cc9283-S1.PDF]

Table 3. TLR4 and injury responsive (TIR) genes list.

Probe Set ID	Gene Symbol	Gene Title
202169_s_at	AASDHPPT	aminoadipate-semialdehyde dehydrogenase-phosphopantetheinyl transferase
205986_at	AATK	apoptosis-associated tyrosine kinase
203192_at	ABCB6	ATP-binding cassette, sub-family B (MDR/TAP), member 6
201872_s_at	ABCE1	ATP-binding cassette, sub-family E (OABP), member 1
200965_s_at	ABLIM1	actin binding LIM protein 1
201630_s_at	ACP1	acid phosphatase 1, soluble
200974_at	ACTA2	actin, alpha 2, smooth muscle, aorta
200720_s_at	ACTR1A	ARP1 actin-related protein 1 homolog A, centractin alpha (yeast)
205260_s_at	ACYP1	acylphosphatase 1, erythrocyte (common) type
201753_s_at	ADD3	adducin 3 (gamma)
202912_at	ADM	adrenomedullin
221761_at	ADSS	adenylosuccinate synthase
206820_at	AGFG2	ArfGAP with FG repeats 2
201675_at	AKAP1	A kinase (PRKA) anchor protein 1
203156_at	AKAP11	A kinase (PRKA) anchor protein 11
205771_s_at	AKAP7	A kinase (PRKA) anchor protein 7
205633_s_at	ALAS1	aminolevulinate, delta-, synthase 1
203608_at	ALDH5A1	aldehyde dehydrogenase 5 family, member A1
205477_s_at	AMBP	alpha-1-microglobulin/bikunin precursor
204976_s_at	AMMECR1	Alport syndrome, mental retardation,
220989_s_at	AMN	amnionless homolog (mouse)
200098_s_at	ANAPC5	anaphase promoting complex subunit 5
205639_at	AOAH	acyloxyacyl hydrolase (neutrophil)
202399_s_at	AP3S2	adaptor-related protein complex 3, sigma 2 subunit
221013_s_at	APOL2	apolipoprotein L, 2
216620_s_at	ARHGEF10	Rho guanine nucleotide exchange factor (GEF) 10
202092_s_at	ARL2BP	ADP-ribosylation factor-like 2 binding protein
218150_at	ARL5A	ADP-ribosylation factor-like 5A
206129_s_at	ARSB	arylsulfatase B
205784_x_at	ARVCF	armadillo repeat gene deletes in velocardiofacial syndrome
205047_s_at	ASNS	asparagine synthetase
212672_at	ATM	ataxia telangiectasia mutated

Table 3. TLR4 and injury responsive (TIR) genes list.

213738_s_at	ATP5A1		ATP synthase, H+ transporting, mitochondrial F1 complex, alpha subunit 1, cardiac muscle
201806_s_at	ATXN2L		ataxin 2-like
209413_at	B4GALT2		UDP-Gal:betaGlcNAc beta 1,4- galactosyltransferase, polypeptide 2
212876_at	B4GALT4		UDP-Gal:betaGlcNAc beta 1,4- galactosyltransferase, polypeptide 4
221234_s_at	BACH2		BTB and CNC homology 1, basic leucine zipper transcription factor 2
205965_at	BATF		basic leucine zipper transcription factor, ATF-like
219528_s_at	BCL11B		B-cell CLL/lymphoma 11B (zinc finger protein)
218285_s_at	BDH2		3-hydroxybutyrate dehydrogenase, type 2
206956_at	BGLAP		bone gamma-carboxyglutamate (gla) protein
210538_s_at	BIRC3		baculoviral IAP repeat-containing 3
202265_at	BMI1		BMI1 polycomb ring finger oncogene
207595_s_at	BMP1		bone morphogenetic protein 1
221454_at	BOK		BCL2-related ovarian killer
205715_at	BST1		bone marrow stromal cell antigen 1
211939_x_at	BTF3		basic transcription factor 3
205690_s_at	BUD31		BUD31 homolog (S. cerevisiae)
64432_at	C12orf47		chromosome 12 open reading frame 47
205781_at	C16orf7		chromosome 16 open reading frame 7
211563_s_at	C19orf2		chromosome 19 open reading frame 2
203052_at	C2		complement component 2
217835_x_at	C20orf24		chromosome 20 open reading frame 24
206656_s_at	C20orf3		chromosome 20 open reading frame 3
221158_at	C21orf66		chromosome 21 open reading frame 66
204968_at	C6orf47		chromosome 6 open reading frame 47
209301_at	CA2		carbonic anhydrase II
204811_s_at	CACNA2D2		calcium channel, voltage-dependent, alpha 2/delta subunit 2
219714_s_at	CACNA2D3		calcium channel, voltage-dependent, alpha 2/delta subunit 3
210244_at	CAMP		cathelicidin antimicrobial peptide
203356_at	CAPN7		calpain 7
211208_s_at	CASK		calcium/calmodulin-dependent serine protein kinase (MAGUK family)
222201_s_at	CASP8AP2		caspase 8 associated protein 2
209682_at	CBLB		Cas-Br-M (murine) ecotropic retroviral transforming sequence b
200953_s_at	CCND2		cyclin D2
204826_at	CCNF		cyclin F
208304_at	CCR3		chemokine (C-C motif) receptor 3
206337_at	CCR7		chemokine (C-C motif) receptor 7

Table 3. TLR4 and injury responsive (TIR) genes list.

219669_at	CD177		CD177 molecule
205831_at	CD2		CD2 molecule
207315_at	CD226		CD226 molecule
206545_at	CD28		CD28 molecule
206804_at	CD3G		CD3g molecule, gamma (CD3-TCR complex)
200663_at	CD63		CD63 molecule
209795_at	CD69		CD69 molecule
202717_s_at	CDC16		cell division cycle 16 homolog (S. cerevisiae)
207318_s_at	CDC2L5		cell division cycle 2-like 5 (cholinesterase-related cell division controller)
206824_at	CES4		carboxylesterase 4-like
203953_s_at	CLDN3		claudin 3
208925_at	CLDND1		claudin domain containing 1
220132_s_at	CLEC2D		C-type lectin domain family 2, member D
218250_s_at	CNOT7		CCR4-NOT transcription complex, subunit 7
218142_s_at	CRBN		cereblon
202979_s_at	CREBZF		CREB/ATF bZIP transcription factor
219939_s_at	CSDE1		cold shock domain containing E1, RNA-binding
210140_at	CST7		cystatin F (leukocystatin)
203758_at	CTSO		cathepsin O
210816_s_at	CYB561		cytochrome b-561
200932_s_at	DCTN2		dynactin 2 (p50)
214909_s_at	DDAH2		dimethylarginine dimethylaminohydrolase 2
208895_s_at	DDX18		DEAD (Asp-Glu-Ala-Asp) box polypeptide 18
201386_s_at	DHX15		DEAH (Asp-Glu-Ala-His) box polypeptide 15
204008_at	DNAL4		dynein, axonemal, light chain 4
207192_at	DNASE1L2		deoxyribonuclease I-like 2
201697_s_at	DNMT1		DNA (cytosine-5-)-methyltransferase 1
204794_at	DUSP2		dual specificity phosphatase 2
202969_at	DYRK2		dual-specificity tyrosine-(Y)-phosphorylation regulated kinase 2
218660_at	DYSF		dysferlin, limb girdle muscular dystrophy 2B (autosomal recessive)
201749_at	ECE1		endothelin converting enzyme 1
206559_x_at	EEF1A1		eukaryotic translation elongation factor 1 alpha 1
218825_at	EGFL7		EGF-like-domain, multiple 7
208697_s_at	EIF3E		eukaryotic translation initiation factor 3, subunit E
200912_s_at	EIF4A2		eukaryotic translation initiation factor 4A, isoform 2
206338_at	ELAVL3		ELAV (embryonic lethal, abnormal vision, Drosophila)-like 3 (Hu antigen C)

Table 3. TLR4 and injury responsive (TIR) genes list.

204143_s_at	ENOSF1	enolase superfamily member 1
202894_at	EPHB4	EPH receptor B4
207541_s_at	EXOSC10	exosome component 10
201995_at	EXT1	exostoses (multiple) 1
204714_s_at	F5	coagulation factor V (proaccelerin, labile factor)
202535_at	FADD	Fas (TNFRSF6)-associated via death domain
221601_s_at	FAIM3	Fas apoptotic inhibitory molecule 3
206848_at	FAM36A /// HOXA7	family with sequence similarity 36, member A /// homeobox A7
204232_at	FCER1G	Fc fragment of IgE, high affinity I, receptor for; gamma polypeptide
216950_s_at	FCGR1A /// FCGR	Fc fragment of IgG, high affinity Ia, receptor (CD64)
221385_s_at	FFAR3	free fatty acid receptor 3
210607_at	FLT3LG	fms-related tyrosine kinase 3 ligand
206371_at	FOLR3	folate receptor 3 (gamma)
204299_at	FUSIP1	FUS interacting protein (serine/arginine-rich) 1
210105_s_at	FYN	FYN oncogene related to SRC, FGR, YES
207574_s_at	GADD45B	growth arrest and DNA-damage-inducible, beta
210446_at	GATA1	GATA binding protein 1 (globin transcription factor 1)
209604_s_at	GATA3	GATA binding protein 3
202270_at	GBP1	guanylate binding protein 1, interferon-inducible, 67kDa
207153_s_at	GLMN	glomulin, FKBP associated protein
35820_at	GM2A	GM2 ganglioside activator
218458_at	GMCL1	germ cell-less homolog 1 (Drosophila)
205349_at	GNA15	guanine nucleotide binding protein (G protein), alpha 15 (Gq class)
200651_at	GNB2L1	guanine nucleotide binding protein (G protein), beta polypeptide 2-like 1
37145_at	GNLY	granulysin
208519_x_at	GNRH2	gonadotropin-releasing hormone 2
210640_s_at	GPER	G protein-coupled estrogen receptor 1
205419_at	GPR183	G protein-coupled receptor 183
212070_at	GPR56	G protein-coupled receptor 56
209409_at	GRB10	growth factor receptor-bound protein 10
218343_s_at	GTF3C3	general transcription factor IIIC, polypeptide 3, 102kDa
211275_s_at	GYG1	glycogenin 1
205488_at	GZMA	granzyme A (granzyme 1, cytotoxic T-lymphocyte-associated serine esterase 3)
210164_at	GZMB	granzyme B (granzyme 2, cytotoxic T-lymphocyte-associated serine esterase 1)
206666_at	GZMK	granzyme K (granzyme 3; tryptase II)
208569_at	HIST1H2AB	histone cluster 1, H2ab

Table 3. TLR4 and injury responsive (TIR) genes list.

214522_x_at	HIST1H2AD /// HIS	histone cluster 1, H2ad /// histone cluster 1, H3d
214472_at	HIST1H2AD /// HIS	histone cluster 1, H2ad /// histone cluster 1, H3d
214634_at	HIST1H4B	Histone cluster 1, H4b
208076_at	HIST1H4D	histone cluster 1, H4d
208551_at	HIST1H4G	histone cluster 1, H4g
205936_s_at	HK3	hexokinase 3 (white cell)
202983_at	HLTF	helicase-like transcription factor
214438_at	HLX	H2.0-like homeobox
218152_at	HMG20A	high-mobility group 20A
209786_at	HMGN4	high mobility group nucleosomal binding domain 4
217755_at	HN1	hematological and neurological expressed 1
200016_x_at	HNRNPA1	heterogeneous nuclear ribonucleoprotein A1
221919_at	HNRNPA1 /// LOC	heterogeneous nuclear ribonucleoprotein A1 /// hypothetical LOC728844
201031_s_at	HNRNPH1	heterogeneous nuclear ribonucleoprotein H1 (H)
201406_at	HNRNPH2 /// RPL	heterogeneous nuclear ribonucleoprotein H2 (H') /// ribosomal protein L36a ///
208766_s_at	HNRNPR	heterogeneous nuclear ribonucleoprotein R
209067_s_at	HNRPDL	heterogeneous nuclear ribonucleoprotein D-like
204544_at	HPS5	Hermansky-Pudlak syndrome 5
219212_at	HSPA14	heat shock 70kDa protein 14
201565_s_at	ID2	inhibitor of DNA binding 2, dominant negative helix-loop-helix protein
201631_s_at	IER3	immediate early response 3
215712_s_at	IGFALS	insulin-like growth factor binding protein, acid labile subunit
204773_at	IL11RA	interleukin 11 receptor, alpha
207844_at	IL13	interleukin 13
212657_s_at	IL1RN	interleukin 1 receptor antagonist
205291_at	IL2RB	interleukin 2 receptor, beta
205798_at	IL7R	interleukin 7 receptor
204169_at	IMPDH1	IMP (inosine monophosphate) dehydrogenase 1
205258_at	INHBB	inhibin, beta B
213416_at	ITGA4	integrin, alpha 4 (antigen CD49D, alpha 4 subunit of VLA-4 receptor)
216331_at	ITGA7	integrin, alpha 7
211339_s_at	ITK	IL2-inducible T-cell kinase
206842_at	KCND1	potassium voltage-gated channel, Shal-related subfamily, member 1
207635_s_at	KCNH1	potassium voltage-gated channel, subfamily H (eag-related), member 1
209661_at	KIFC3	kinesin family member C3
211410_x_at	KIR2DL5A	killer cell immunoglobulin-like receptor, two domains, long cytoplasmic tail, 5A

Table 3. TLR4 and injury responsive (TIR) genes list.

211532_x_at	KIR2DS1 /// KIR2D	killer cell immunoglobulin-like receptor, two domains, short cytoplasmic tail, 1 /tail, 2 / tail, 4
208203_x_at	KIR2DS5	killer cell immunoglobulin-like receptor, two domains, short cytoplasmic tail, 5
207314_x_at	KIR3DL2 /// LOC72	killer cell immunoglobulin-like receptor, three domains, long cytoplasmic tail, 2 ///
217906_at	KLHDC2	kelch domain containing 2
221221_s_at	KLHL3	kelch-like 3 (Drosophila)
214470_at	KLRB1	killer cell lectin-like receptor subfamily B, member 1
206785_s_at	KLRC1	killer cell lectin-like receptor subfamily C, member 1 /// member 2
207723_s_at	KLRC3	killer cell lectin-like receptor subfamily C, member 3
207795_s_at	KLRD1	killer cell lectin-like receptor subfamily D, member 1
220646_s_at	KLRF1	killer cell lectin-like receptor subfamily F, member 1
210288_at	KLRG1	killer cell lectin-like receptor subfamily G, member 1
205821_at	KLRK1	killer cell lectin-like receptor subfamily K, member 1
202020_s_at	LANCL1	LanC lantibiotic synthetase component C-like 1 (bacterial)
204891_s_at	LCK	lymphocyte-specific protein tyrosine kinase
201030_x_at	LDHB	lactate dehydrogenase B
221558_s_at	LEF1	lymphoid enhancer-binding factor 1
206230_at	LHX1	LIM homeobox 1
214035_x_at	LOC399491	GPS, PLAT and transmembrane domain-containing protein
216902_s_at	LOC653390 /// LO	RRN3 RNA polymerase I transcription factor homolog (S. cerevisiae) pseudogene ///
204381_at	LRP3	low density lipoprotein receptor-related protein 3
210128_s_at	LTB4R	leukotriene B4 receptor
220130_x_at	LTB4R2	leukotriene B4 receptor 2
204970_s_at	MAFG	v-maf musculoaponeurotic fibrosarcoma oncogene homolog G (avian)
206571_s_at	MAP4K4	mitogen-activated protein kinase kinase kinase kinase 4
205050_s_at	MAPK8IP2	mitogen-activated protein kinase 8 interacting protein 2
205819_at	MARCO	macrophage receptor with collagenous structure
200626_s_at	MATR3	matrin 3
218440_at	MCCC1	methylcrotonoyl-Coenzyme A carboxylase 1 (alpha)
203497_at	MED1	mediator complex subunit 1
209199_s_at	MEF2C	myocyte enhancer factor 2C
209861_s_at	METAP2	methionyl aminopeptidase 2
206560_s_at	MIA	melanoma inhibitory activity
201285_at	MKRN1	makorin ring finger protein 1
203936_s_at	MMP9	matrix metallopeptidase 9 (gelatinase B, 92kDa gelatinase, 92kDa type IV collagenase)
212508_at	MOAP1	modulator of apoptosis 1
202974_at	MPP1	membrane protein, palmitoylated 1, 55kDa

Table 3. TLR4 and injury responsive (TIR) genes list.

210356_x_at	MS4A1	membrane-spanning 4-domains, subfamily A, member 1
209421_at	MSH2	mutS homolog 2, colon cancer, nonpolyposis type 1 (E. coli)
218773_s_at	MSRB2	methionine sulfoxide reductase B2
203433_at	MTHFS	5,10-methenyltetrahydrofolate synthetase (5-formyltetrahydrofolate cyclo-ligase)
219822_at	MTRF1	mitochondrial translational release factor 1
210386_s_at	MTX1	metaxin 1
202431_s_at	MYC	v-myc myelocytomatosis viral oncogene homolog (avian)
209124_at	MYD88	myeloid differentiation primary response gene (88)
212082_s_at	MYL6	myosin, light chain 6, alkali, smooth muscle and non-muscle
212462_at	MYST4	MYST histone acetyltransferase (monocytic leukemia) 4
200735_x_at	NACA	nascent polypeptide-associated complex alpha subunit
218189_s_at	NANS	N-acetylneuraminic acid synthase
208754_s_at	NAP1L1	nucleosome assembly protein 1-like 1
200610_s_at	NCL	nucleolin
200632_s_at	NDRG1	N-myc downstream regulated 1
201304_at	NDUFA5	NADH dehydrogenase (ubiquinone) 1 alpha subcomplex, 5, 13kDa
203413_at	NELL2	NEL-like 2 (chicken)
218127_at	NFYB	nuclear transcription factor Y, beta
206814_at	NGF	nerve growth factor (beta polypeptide)
202891_at	NIT1	nitrilase 1
205004_at	NKRF	NFKB repressing factor
215338_s_at	NKTR	natural killer-tumor recognition sequence
219726_at	NLGN3	neuroligin 3
205005_s_at	NMT2	N-myristoyltransferase 2
209798_at	NPAT	nuclear protein, ataxia-telangiectasia locus
200063_s_at	NPM1	nucleophosmin (nucleolar phosphoprotein B23, numatrin)
203814_s_at	NQO2	NAD(P)H dehydrogenase, quinone 2
205259_at	NR3C2	nuclear receptor subfamily 3, group C, member 2
202600_s_at	NRIP1	nuclear receptor interacting protein 1
203939_at	NT5E	5'-nucleotidase, ecto (CD73)
219708_at	NT5M	5',3'-nucleotidase, mitochondrial
217802_s_at	NUCKS1	nuclear casein kinase and cyclin-dependent kinase substrate 1
202155_s_at	NUP214	nucleoporin 214kDa
202073_at	OPTN	optineurin
219475_at	OSGIN1	oxidative stress induced growth inhibitor 1
208717_at	OXA1L	oxidase (cytochrome c) assembly 1-like

Table 3. TLR4 and injury responsive (TIR) genes list.

214615_at	P2RY10	purinergic receptor P2Y, G-protein coupled, 10
215157_x_at	PABPC1	poly(A) binding protein, cytoplasmic 1
208113_x_at	PABPC3	poly(A) binding protein, cytoplasmic 3
202760_s_at	PALM2-AKAP2	PALM2-AKAP2 readthrough transcript
211867_s_at	PCDHA10	protocadherin alpha 10
202174_s_at	PCM1	pericentriolar material 1
203660_s_at	PCNT	pericentrin
207634_at	PDCD1	programmed cell death 1
206444_at	PDE1B	phosphodiesterase 1B, calmodulin-dependent
221957_at	PDK3	pyruvate dehydrogenase kinase, isozyme 3
202464_s_at	PFKFB3	6-phosphofructo-2-kinase/fructose-2,6-biphosphatase 3
207384_at	PGLYRP1	peptidoglycan recognition protein 1
208491_s_at	PGM5	phosphoglucomutase 5
204746_s_at	PICK1	protein interacting with PRKCA 1
221689_s_at	PIGP	phosphatidylinositol glycan anchor biosynthesis, class P
208277_at	PITX3	paired-like homeodomain 3
219024_at	PLEKHA1	pleckstrin homology domain containing, family A (phosphoinositide binding specific)
219700_at	PLXDC1	plexin domain containing 1
220923_s_at	PNMA3	paraneoplastic antigen MA3
203622_s_at	PNO1	partner of NOB1 homolog (S. cerevisiae)
205811_at	POLG2	polymerase (DNA directed), gamma 2, accessory subunit
202466_at	POLS	polymerase (DNA directed) sigma
201293_x_at	PPIA	peptidylprolyl isomerase A (cyclophilin A)
200726_at	PPP1CC	protein phosphatase 1, catalytic subunit, gamma isoform
202165_at	PPP1R2	protein phosphatase 1, regulatory (inhibitor) subunit 2
207830_s_at	PPP1R8	protein phosphatase 1, regulatory (inhibitor) subunit 8
203338_at	PPP2R5E	protein phosphatase 2, regulatory subunit B', epsilon isoform
220654_at	PPY2	pancreatic polypeptide 2
214617_at	PRF1	perforin 1 (pore forming protein)
202742_s_at	PRKACB	protein kinase, cAMP-dependent, catalytic, beta
209678_s_at	PRKCI	protein kinase C, iota
210039_s_at	PRKCQ	protein kinase C, theta
221443_x_at	PRLH	prolactin releasing hormone
207291_at	PRRG4	proline rich Gla (G-carboxyglutamic acid) 4 (transmembrane)
202458_at	PRSS23	protease, serine, 23
205961_s_at	PSIP1	PC4 and SFRS1 interacting protein 1

Table 3. TLR4 and injury responsive (TIR) genes list.

209337_at	PSIP1	PC4 and SFRS1 interacting protein 1
218967_s_at	PTER	phosphotriesterase related
200627_at	PTGES3	prostaglandin E synthase 3 (cytosolic)
205171_at	PTPN4	protein tyrosine phosphatase, non-receptor type 4 (megakaryocyte)
204020_at	PURA	purine-rich element binding protein A
203149_at	PVRL2	poliovirus receptor-related 2 (herpesvirus entry mediator B)
201606_s_at	PWP1	PWP1 homolog (S. cerevisiae)
201482_at	QSOX1	quiescin Q6 sulfhydryl oxidase 1
202252_at	RAB13	RAB13, member RAS oncogene family
204214_s_at	RAB32	RAB32, member RAS oncogene family
219151_s_at	RABL2A /// RABL2	RAB, member of RAS oncogene family-like 2A /// 2B
221830_at	RAP2A	RAP2A, member of RAS oncogene family
203749_s_at	RARA	retinoic acid receptor, alpha
212706_at	RASA4	RAS p21 protein activator 4 pseudogene /// RAS p21 protein activator 4
205590_at	RASGRP1	RAS guanyl releasing protein 1 (calcium and DAG-regulated)
208319_s_at	RBM3	RNA binding motif (RNP1, RRM) protein 3
201967_at	RBM6	RNA binding motif protein 6
213520_at	RECQL4	RecQ protein-like 4
205645_at	REPS2	RALBP1 associated Eps domain containing 2
220570_at	RETN	resistin
203823_at	RGS3	regulator of G-protein signaling 3
205211_s_at	RIN1	Ras and Rab interactor 1
201785_at	RNASE1	ribonuclease, RNase A family, 1 (pancreatic)
219104_at	RNF141	ring finger protein 141
202683_s_at	RNMT	RNA (guanine-7-) methyltransferase
205806_at	ROM1	retinal outer segment membrane protein 1
210479_s_at	RORA	RAR-related orphan receptor A
201528_at	RPA1	replication protein A1, 70kDa
200036_s_at	RPL10A	ribosomal protein L10a
200010_at	RPL11	ribosomal protein L11
200074_s_at	RPL14	ribosomal protein L14 /// ribosomal protein L14 pseudogene 1
221476_s_at	RPL15	ribosomal protein L15
200038_s_at	RPL17	ribosomal protein L17
200029_at	RPL19	ribosomal protein L19
200012_x_at	RPL21	ribosomal protein L21
208768_x_at	RPL22	ribosomal protein L22

Table 3. TLR4 and injury responsive (TIR) genes list.

200888_s_at	RPL23	ribosomal protein L23
208825_x_at	RPL23A	ribosomal protein L23a
200013_at	RPL24	ribosomal protein L24
203034_s_at	RPL27A	ribosomal protein L27a
200062_s_at	RPL30	ribosomal protein L30
200026_at	RPL34	ribosomal protein L34
200002_at	RPL35	ribosomal protein L35
200092_s_at	RPL37	ribosomal protein L37
208695_s_at	RPL39	ribosomal protein L39
200089_s_at	RPL4	ribosomal protein L4
200937_s_at	RPL5	ribosomal protein L5
200034_s_at	RPL6	ribosomal protein L6
200717_x_at	RPL7	ribosomal protein L7
200032_s_at	RPL9	ribosomal protein L9
200909_s_at	RPLP2	ribosomal protein, large, P2
213377_x_at	RPS12	ribosomal protein S12
200018_at	RPS13	ribosomal protein S13
200781_s_at	RPS15A	ribosomal protein S15a
201049_s_at	RPS18	ribosomal protein S18
200834_s_at	RPS21	ribosomal protein S21
200926_at	RPS23	ribosomal protein S23
200061_s_at	RPS24	ribosomal protein S24
200091_s_at	RPS25	ribosomal protein S25
200741_s_at	RPS27	ribosomal protein S27
200017_at	RPS27A	ribosomal protein S27a
201094_at	RPS29	ribosomal protein S29
200099_s_at	RPS3A	ribosomal protein S3A
200933_x_at	RPS4X	ribosomal protein S4, X-linked
200081_s_at	RPS6	ribosomal protein S6
203379_at	RPS6KA1	ribosomal protein S6 kinase, 90kDa, polypeptide 1
200082_s_at	RPS7 /// RPS7P11	ribosomal protein S7 /// ribosomal protein S7 pseudogene 11
200858_s_at	RPS8	ribosomal protein S8
217915_s_at	RSL24D1	ribosomal L24 domain containing 1
34408_at	RTN2	reticulon 2
204197_s_at	RUNX3	runt-related transcription factor 3
205863_at	S100A12	S100 calcium binding protein A12

Table 3. TLR4 and injury responsive (TIR) genes list.

204642_at	S1PR1	sphingosine-1-phosphate receptor 1
200051_at	SART1	squamous cell carcinoma antigen recognized by T cells
203408_s_at	SATB1	SATB homeobox 1
210364_at	SCN2B	sodium channel, voltage-gated, type II, beta
205241_at	SCO2	SCO cytochrome oxidase deficient homolog 2 (yeast)
201339_s_at	SCP2	sterol carrier protein 2
202542_s_at	SCYE1	small inducible cytokine subfamily E, member 1 (endothelial monocyte-activating)
203090_at	SDF2	stromal cell-derived factor 2
220778_x_at	SEMA6B	sema domain, transmembrane domain (TM), and cytoplasmic domain, (semaphorin) 6B
211429_s_at	SERPINA1	serpin peptidase inhibitor, clade A (alpha-1 antiproteinase, antitrypsin), member 1
218346_s_at	SESN1	sestrin 1
200686_s_at	SFRS11	splicing factor, arginine/serine-rich 11
221268_s_at	SGPP1	sphingosine-1-phosphate phosphatase 1
210116_at	SH2D1A	SH2 domain protein 1A
202896_s_at	SIRPA	signal-regulatory protein alpha
207974_s_at	SKP1	S-phase kinase-associated protein 1
210423_s_at	SLC11A1	solute carrier family 11 (proton-coupled divalent metal ion transporters), member 1
207567_at	SLC13A2	solute carrier family 13 (sodium-dependent dicarboxylate transporter), member 2
211576_s_at	SLC19A1	solute carrier family 19 (folate transporter), member 1
205097_at	SLC26A2	solute carrier family 26 (sulfate transporter), member 2
202499_s_at	SLC2A3	solute carrier family 2 (facilitated glucose transporter), member 3
218237_s_at	SLC38A1	solute carrier family 38, member 1
213164_at	SLC5A3	solute carrier family 5 (sodium/myo-inositol cotransporter), member 3
202219_at	SLC6A8	solute carrier family 6 (neurotransmitter transporter, creatine), member 8
203579_s_at	SLC7A6	solute carrier family 7 (cationic amino acid transporter, y+ system), member 6
203021_at	SLPI	secretory leukocyte peptidase inhibitor
211988_at	SMARCE1	SWI/SNF related, matrix associated, actin dependent regulator of chromatin
203852_s_at	SMN1 /// SMN2	survival of motor neuron 1, telomeric /// survival of motor neuron 2, centromeric
205300_s_at	SNRNP35	small nuclear ribonucleoprotein 35kDa (U11/U12)
219257_s_at	SPHK1	sphingosine kinase 1
218638_s_at	SPON2	spondin 2, extracellular matrix protein
217995_at	SQRDL	sulfide quinone reductase-like (yeast)
201273_s_at	SRP9	signal recognition particle 9kDa
201225_s_at	SRRM1	serine/arginine repetitive matrix 1
201138_s_at	SSB	Sjogren syndrome antigen B (autoantigen La)
208666_s_at	ST13	suppression of tumorigenicity 13 (colon carcinoma) (Hsp70 interacting protein)

Table 3. TLR4 and injury responsive (TIR) genes list.

205346_at	ST3GAL2	ST3 beta-galactoside alpha-2,3-sialyltransferase 2
203759_at	ST3GAL4	ST3 beta-galactoside alpha-2,3-sialyltransferase 4
220059_at	STAP1	signal transducing adaptor family member 1
AFFX-HUMISG	STAT1	signal transducer and activator of transcription 1, 91kDa
206118_at	STAT4	signal transducer and activator of transcription 4
207601_at	SULT1B1	sulfotransferase family, cytosolic, 1B, member 1
217833_at	SYNCRIP	synaptotagmin binding, cytoplasmic RNA interacting protein
208048_at	TACR1	tachykinin receptor 1
202840_at	TAF15	TAF15 RNA polymerase II, TATA box binding protein (TBP)-associated factor, 68kDa
204986_s_at	TAOK2	TAO kinase 2
202813_at	TARBP1	TAR (HIV-1) RNA binding protein 1
220634_at	TBX4	T-box 4
202396_at	TCERG1	transcription elongation regulator 1
203753_at	TCF4	transcription factor 4
203449_s_at	TERF1	telomeric repeat binding factor (NIMA-interacting) 1
204731_at	TGFBR3	transforming growth factor, beta receptor III
204064_at	THOC1	THO complex 1
209418_s_at	THOC5	THO complex 5
217847_s_at	THRAP3	thyroid hormone receptor associated protein 3
219477_s_at	THSD1 /// THSD1P	thrombospondin, type I, domain containing 1 /// pseudogene
203167_at	TIMP2	TIMP metallopeptidase inhibitor 2
203437_at	TMEM11	transmembrane protein 11
200847_s_at	TMEM66	transmembrane protein 66
210260_s_at	TNFAIP8	tumor necrosis factor, alpha-induced protein 8
202807_s_at	TOM1	target of myb1 (chicken)
200662_s_at	TOMM20	translocase of outer mitochondrial membrane 20 homolog (yeast)
203421_at	TP53I11	tumor protein p53 inducible protein 11
210609_s_at	TP53I3	tumor protein p53 inducible protein 3
216485_s_at	TPSAB1	tryptase alpha/beta 1
205599_at	TRAF1	TNF receptor-associated factor 1
204352_at	TRAF5	TNF receptor-associated factor 5
35254_at	TRAFD1	TRAF-type zinc finger domain containing 1
208662_s_at	TTC3	tetratricopeptide repeat domain 3
206828_at	TXK	TXK tyrosine kinase
218011_at	UBL5	ubiquitin-like 5
203234_at	UPP1	uridine phosphorylase 1

Table 3. TLR4 and injury responsive (TIR) genes list.

218386_x_at	USP16	ubiquitin specific peptidase 16
206624_at	USP9Y	ubiquitin specific peptidase 9, Y-linked
209486_at	UTP3	UTP3, small subunit (SSU) processome component, homolog (S. cerevisiae)
218715_at	UTP6	UTP6, small subunit (SSU) processome component, homolog (yeast)
208780_x_at	VAPA	VAMP (vesicle-associated membrane protein)-associated protein A, 33kDa
204022_at	WWP2	WW domain containing E3 ubiquitin protein ligase 2
213081_at	ZBTB22	zinc finger and BTB domain containing 22
218078_s_at	ZDHHC3	zinc finger, DHHC-type containing 3
201368_at	ZFP36L2	zinc finger protein 36, C3H type-like 2
202136_at	ZMYND11	zinc finger, MYND domain containing 11
219571_s_at	ZNF12	zinc finger protein 12
206931_at	ZNF141	zinc finger protein 141
206416_at	ZNF205	zinc finger protein 205
203707_at	ZNF263	zinc finger protein 263
219228_at	ZNF331	zinc finger protein 331
206059_at	ZNF91	zinc finger protein 91
